# Understudied Hyperphosphatemia (Chronic Kidney Disease) Treatment Targets and New Biological Approaches

**DOI:** 10.3390/medicina59050959

**Published:** 2023-05-16

**Authors:** Ajeeta Anand, Hideki Aoyagi

**Affiliations:** 1 Institute of Life Sciences and Bioengineering, Graduate School of Life and Environmental Sciences, University of Tsukuba, Tsukuba 305-8572, Japan; 2 Institute of Life and Environmental Sciences, University of Tsukuba, Tsukuba 305-8572, Japan

**Keywords:** chronic kidney disorder, hyperphosphatemia, phosphate accumulating organisms, phosphate-controlled diet

## Abstract

Hyperphosphatemia is a secondary disorder of chronic kidney disease that causes vascular calcifications and bone-mineral disorders. As per the US Centers for Disease Control and Prevention, renal damage requires first-priority medical attention for patients with COVID-19; according to a Johns Hopkins Medicine report, SARS-CoV-2 can cause renal damage. Therefore, addressing the research inputs required to manage hyperphosphatemia is currently in great demand. This review highlights research inputs, such as defects in the diagnosis of hyperphosphatemia, flaws in understanding the mechanisms associated with understudied tertiary toxicities, less cited adverse effects of phosphate binders that question their use in the market, socioeconomic challenges of renal treatment and public awareness regarding the management of a phosphate-controlled diet, novel biological approaches (synbiotics) to prevent hyperphosphatemia as safer strategies with potential additional health benefits, and future functional food formulations to enhance the quality of life. We have not only introduced our contributions to emphasise the hidden aspects and research gaps in comprehending hyperphosphatemia but also suggested new research areas to strengthen approaches to prevent hyperphosphatemia in the near future.

## 1. Introduction

Chronic kidney disease (CKD) is a common disorder, with 10% of the world population suffering from it and millions of deaths reported every year due to the unavailability of an affordable treatment [1]. Hyperphosphatemia is a prevalent comorbidity of CKD, affecting 50–74% of patients with renal disorders [2]. Kidney function anomalies result in phosphate imbalance in the body, leading to hyperphosphatemia, a condition known to cause life-threatening complications, including bone mineral imbalances and vascular calcification [3]. When a patient is diagnosed with hyperphosphatemia, phosphate-rich foods are immediately restricted to reduce the dietary-phosphate load, which hampers the quality of life. These dietary restrictions, coupled with the potential side effects of phosphate binders, further exacerbate the already compromised quality of life of patients due to the long and strict dialysis schedule [3].

Our current review highlights the gaps in the knowledge of hyperphosphatemia management that need immediate attention, such as performing research to identify the unheeded sources of phosphate in the daily routine, cross-reactivity of phosphate with food processing reactions and its impact on renal health, accuracy of phosphate estimation in blood samples, practical efficacy of hyperphosphatemia treatment, potential consequences of hyperphosphatemia, and topics that require immediate attention (renal damage caused by heavy metals in cosmetics and SARS-CoV-2; Supplementary Material) for general awareness. The disadvantages of phosphate binders and kidney-associated disorders that have been scarcely addressed are also included in this review.

## 2. Phosphate-Associated Toxicities

It is evident from the literature that high serum phosphate levels are linked to cardiovascular adverse events among non-CKD and CKD populations [4]. Among CKD populations, mortalities in stages 3 and 4 are linked to high serum phosphate concentrations [5], whereas stage 5 (no dialysis) is linked to a high risk of mortality due to increased serum phosphate levels of 4.71 mg/dL [6]. In the largest study reported at the Veterans Affairs Medical Centers (n 1/4 3490) by Kestenbaum et al., the mortality risk for patients with CKD was linearly associated with the serum phosphate threshold of 3.5 mg/dL (1.13 mmoL/L) [7].

### 2.1. Calcification

In hyperphosphatemia, high levels of intact parathyroid hormone (iPTH) and fibroblast growth factor (FGF)-23 with reduced vitamin D concentration cause calcium release from bones, which triggers the calcification of vasculatures and weakens the bones. High iPTH levels in hyperphosphatemia are documented in reports related to calcification issues [8,9]. However, these reports did not explore the FGF23 and vitamin D levels to clarify the role of iPTH-related calcifications in renal-based hyperphosphatemia models. In contrast, another report revealed FGF23 as a regulatory factor for cardiac hypertrophy development in a renal disorder-based model, although it did not document iPTH and vitamin D levels [10].

Surprisingly, hypocalcemia is generally reported in hyperphosphatemia [11], albeit when there is frequent calcification of vascular smooth muscle cells. The probable reason for this is calcium gradient formation by bones via local transport phenomena at sites near the vascular organs rather than low systemic calcium levels or hypocalcemia [12]. Bones are calcium reservoirs, which may release a high concentration of free calcium in hyperphosphatemia. This high calcium concentration near vascular tissues and organs leads to calcification issues, rather than calcium in the blood or from the diet.

### 2.2. Local pH

The kidneys regulate electrolyte balance in the blood. However, their capability for bicarbonate reabsorption declines with damage, although the acidogenesis in CKD remains the same and leads to acidosis [13]. Subsequently, intrarenal NH_3_/NH_4_^+^ increases and activates the complement pathway, which causes tubulointerstitial inflammation. This acidic condition exacerbates CKD progression [14]. Additionally, metabolic acidosis causes muscle wasting, protein catabolism, bone demineralisation, thyroid disorders, insulin resistance, growth hormone secretion, exacerbation of β2-microglobulin accumulation, and increased mortality [15,16]. Hyperkalemia, metabolic acidosis, and hyperphosphatemia are indicators of an acid-base imbalance that may result in severe disease.

Treatment of patients with CKD with oral alkali (NaHCO_3_ at 22–24 mmol/L) is a prevention strategy to combat acidosis development [17]. However, precautions must be taken to check serum bicarbonate (>26 mmol/L) as it is associated with mortality risk [18]. Electrolyte imbalance can be corrected by dialysis. However, sudden shifts in pH in a short time caused by dialysis may lead to multiple adverse conditions; for example, high blood pH may cause hypoventilation due to reduced functionality of the central respiratory centre, resulting in reduced O_2_ delivery due to vasoconstriction and shifting of the haemoglobin dissociation curve to the left [19]. High blood pH indicates a high concentration of bicarbonate ions that can bind to systemic acids, thereby resulting in CO_2_ accumulation and paradoxical intracellular acidosis with multiple cellular defects [20].

In conclusion, both acidosis and alkalosis are harmful to pH homeostasis. According to the Bohr effect, to correct pH imbalance in a renal damage condition like hyperphosphatemia, acidosis may lead to higher oxygen levels than normal that may cause oxidative damage to tissues and organs, whereas alkalosis may result in an increase in carbon dioxide levels to maintain pH homeostasis [21], which may enhance the severity of renal disorders.

In hyperphosphatemia, metabolic acidosis not only enhances inflammation but also supports phosphate transport for calcification. In the case of metabolic acidosis or low physiological pH, the majority of phosphate ions are favored to be in their monovalent (H_2_PO_4_^−^) ion forms, which may facilitate phosphate-calcium complex formation. However, to facilitate a calcification reaction, acidic local pH is preferred over the systemic effect of metabolic acidosis.

### 2.3. Phosphate Estimation

In hyperphosphatemia, low bone mass contributes to high phosphate levels in the blood, and the resulting phosphate accumulation causes kidney damage; under these circumstances, phosphate equilibrium is re-established with higher concentrations of blood phosphate and other phosphate reservoirs in the body [22]. This condition cannot be treated by dialysis since a high phosphate equilibrium is established in a short time after dialysis rounds [23]. The health hazards of the hyperphosphatemia initiation phase are reported by the Framingham Offspring Cohort study, wherein researchers found that under normal serum phosphate concentrations, adverse cardiovascular events occurred [24] that raised uncertainty about the real estimation of phosphate levels that are responsible for phosphate-related toxicities. In addition, after a phosphate-rich diet, false-positive blood phosphate levels can be observed. Additionally, if we consider fasted blood samples for phosphate estimation, would the measured value be equal or close to the actual value of the toxic phosphate level?

We should consider the other various forms of phosphate present in the body that may contribute to hyperphosphatemia. Additionally, to adequately treat hyperphosphatemia, we should focus on treating the related toxicities; this treatment strategy should be promoted, although there are no proper guidelines issued to treat hyperphosphatemia entirely. With this treatment, we must pay attention to prevention strategies and spread awareness about phosphate sources in our daily routine, as care is always better than cure.

## 3. Hyperphosphatemia and Phosphate in Food

**Figure 1:**
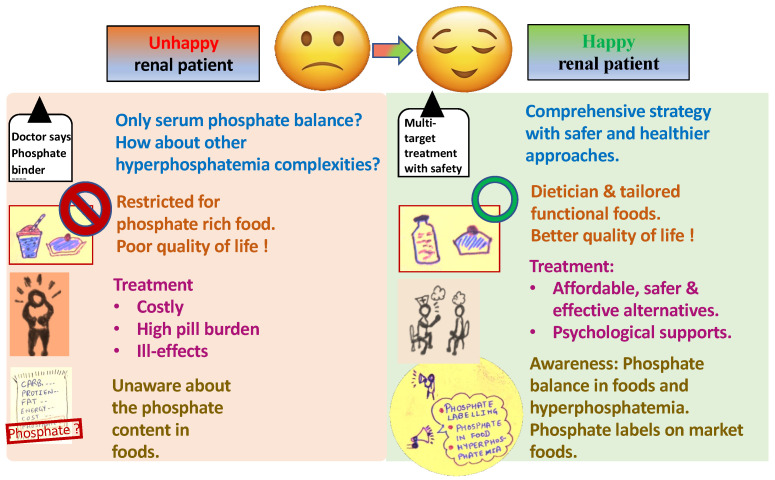
Challenges faced by a renal (hyperphosphatemia) patient and the possible solution.

A patient suffering from renal disorders faces many challenges, such as late diagnosis of associated diseases like hyperphosphatemia and related consequences, poor quality of life, and economic issues, as described in the red part of Figure 1. These problems can be solved by the detection of improved or new markers for hyperphosphatemia diagnosis, development of cost-effective treatment alternatives, diet modification, and following social awareness programs, as shown in the green part of Figure 1. These challenges are described in the following sections with possible solutions.

Preservatives are reported to increase phosphate content in food by as much as 70% [25]; therefore, they are a chief contributor to the postprandial phosphate burden. As per the guidelines of the European Union, sodium phosphate (E 339), potassium phosphate (E 340), calcium phosphate (E 341), salts of orthophosphoric acid diphosphate (E 450), triphosphate (E 451), and polyphosphate (E 452) can be incorporated as preservatives, emulsifying agents, acidity buffers, taste intensifiers, stabilisers, and acidifying agents into food to prevent the growth of microbes. The phosphate content in processed food (including phosphate additives) is reported to be higher than that of natural food due to its high assimilation into the gastrointestinal tract [26]. In addition to preservatives, phosphate additives are also used to prevent the agglomeration of coffee and as a component of the melting salt used for softening cheese [26].

Kemi et al. reported that the consumption of phosphate additive processed cheese considerably increases iPTH levels [27]. In normal renal function, the consumption of a phosphate additive-based diet may result in high levels of FGF-23, osteopontin, and osteocalcin [28]. As per the guidelines of Kidney Disease: Improving Global Outcomes (KDIGO), patients with CKD and bone mineral disorders should not only limit phosphate intake (especially no more than 1 g per day for CKD stage 5 patients) but also undergo proper treatment [29].

Currently, detailed studies are required to understand the effect of the frequency and content of phosphate-rich sources on hyperphosphatemia development at each stage of CKD. In the early stages of CKD, consumption of phosphate-rich food is not associated with premature fatality due to the presence of a healthy kidney function; however, patients on haemodialysis are at a higher risk of mortality with frequent phosphate-rich food consumption [30].

Serum phosphate levels are dependent not only on the phosphate content of consumed food and its digestibility but also on many factors like calcium intake, vitamin D, and the expression of phosphate transporters in intestinal regions [31]. The phosphates present in medications and multivitamin tablets that contribute to 20–150 mg phosphate per supplemental tablet must be investigated for in vivo assimilation studies [32]. Expert dieticians are required to personalise the diet, and address food availability and economic issues with the management of the quality of life of patients with CKD.

### 3.1. Socioeconomic Challenges

Owing to time limitations, lack of cooking skills, and taste preferences, people usually buy marketed foods. It becomes more challenging when the amount of phosphate-based food additives or phosphate content is not mentioned in the marketed foods [33]. The phosphate additives in animal food may intensify the ageing process by accelerating muscle and skin atrophy, thereby resulting in the advancement of CKD and related calcification [34]. Marketed food products have 100% phosphate bioavailability; however, natural food products range between 60 and 80% [35,36]. Thermal processing, such as boiling, is reported to drain out the mineral content of food and assist in the reduction of phosphate content by 35–50% in boiled food [37]. However, it is well-documented that phosphate salts accelerate the formation of Maillard reaction products [38,39,40] (especially monohydrogen phosphate-based salt [41]) during the cooking or processing of foods that later form uremic toxins and overburden the kidney [42,43].

Simple meals with phosphorus additives have 736 mg more phosphorus (41% increment) per day than additive-free foods; however, phosphate additive-free meals are costlier by USD 2.00 per day. Therefore, consumers may prefer lower-priced food and unintentionally acquire the harmful effects of high phosphate consumption [34]. These reports also indicate the socioeconomic imbalance that focuses on the vulnerability of low-income countries to the development of phosphate-related ailments.

### 3.2. Public Education

In a report by Sullivan et al., awareness of phosphate restriction helped to moderately lower the hyperphosphatemia condition in patients with end-stage renal disease [44]. However, a systematic review discovered that the prevention of malnutrition with a reduction in serum phosphate levels in patients with CKD through counselling and raising awareness regarding phosphate-related toxicities and phosphate content in commercial and natural resources is a challenging task [45].

In UK and Finland, products containing sodium chloride are labelled using the colours of a traffic light (i.e., red, yellow, and green marks indicating high, medium, and low content, respectively) to prevent the consumption of high levels of sodium associated with cardiovascular adverse events. Similarly, support for phosphate regulation and the content mentioned on market products must be initiated by medical agencies and governments [26]; consumers must also be concerned and aware of the probable harmful effects of phosphate and develop a habit of checking the phosphate content on the label of processed foods.

## 4. Phosphate Binders in Hyperphosphatemia

Oral phosphate binders work by hindering the intestinal absorption of phosphate and forming an insoluble complex. As per reported studies, two major phosphate binders are currently available in the market: calcium-containing binders and non-calcium-based binders (lanthanum carbonate and aluminium hydroxide). The various advantages and disadvantages of the available phosphate binders are described in Table 1.

### Novel Phosphate Binders

Currently, Sevelamer has drawbacks, including a high dose causing gastrointestinal complications, acidosis, and affordability issues [56]. Another new alternative for phosphate binders is nicotinamide, which regulates hyperphosphatemia by acting negatively on sodium-dependent phosphate transport in renal proximal tubules and the intestine [57]; however, its mechanism of action is yet to be elucidated in detail. In addition, its administration in varied populations resulted in severe gastrointestinal side effects and thrombocytopenia, which restricts its widespread application [58,59]. While tenapanor has been traditionally utilised as a treatment for constipation, recent studies suggest that it may also function as a phosphate binder with a low pill burden and frequency. However, tenapanor can cause nausea and diarrhoea [54]. Ferric citrate and sucroferric oxyhydroxide are two commercially available, iron-based phosphate binders that effectively manage blood phosphate levels and also enhance iron absorption [51]. However, iron-based phosphate binders are relatively expensive and may pose gastrointestinal risks [52].

We conclude that none of the commercially available phosphate binders is ideal or effective, as even the most effective phosphate binders have disadvantages. For example, phosphate binders prescribed to patients with CKD-associated hyperphosphatemia contribute to the pill burden and are costly (USD 750 million globally) [60]. In addition, proper validation of phosphate binders for hyperphosphatemia management with no harmful side effects at clinical levels is still lacking.

The differences in the outcomes of phosphate binder clinical trials could be due to the variation in phosphate exposure, baseline serum phosphate level, population size, and duration. The differences in clinical outcomes have resulted in the spread of misinformation and the wastage of research funds and labour. One of the main reasons for different outcomes is the misinterpretation of statistical values obtained from software data, for example, a *p* value of less than 0.05 is considered significant. This generally prevents the interpretation of other important factors that contribute to the significance of the result. A rational understanding of statistical numbers and comparison with the results in the literature is necessary for laying down a stronger foundation to understand the status of research and future interests.

## 5. Advanced CKD-Associated Understudied Diseases

### 5.1. Pulmonary and Cardiac Irregularities

Pulmonary dysfunction, which is common among patients with end-stage renal disease, is marked by structural and functional cardiac aberrations. The main cause of pulmonary dysfunction with renal failure is the long-term accumulation of uremic toxins that create more physiologically hazardous conditions like the imbalance between acidosis and alkalosis (or hyperphosphatemia). Hyperparathyroidism in patients with CKD resulted in pulmonary calcification, hypertension, and right ventricular hypertrophy as secondary effects [61]. As reported in the literature, the calcium content in the lungs was higher in cases of renal failure (7656 ± 1657 mg/kg dry weight) than in cases of parathyroidectomy-induced renal failure in dogs (1057 ± 117 mg/kg dry weight). According to the same study, the pressure in the right ventricular valve in the dogs with renal failure was higher (30 and 45 [36 ± 2.1] mmHg) than in the parathyroidectomy-induced renal failure dogs (15 and 25 [22 ± 2.0] mmHg). The gas diffusion capacity was also lower (between 2 and 4 mL/min/mmHg) in dogs with renal failure than in normal dogs (from 11.8 to 19.1 mL/min/mmHg) [62]. In a clinical trial, patients with renal disease were reported to struggle with pulmonary venous congestion (15.79%), pleural effusions (10.52%), pericardial effusion (15.79%), and cardiomegaly (15.79%) [63]. Fauber et al. established the direct relation between pulmonary calcification and functional aberrations in pulmonary function in renal failure cases [64]. However, data on pulmonary dysfunction in CKD is still scarce.

### 5.2. Restless Leg Syndrome

Takaki et al. concluded that hyperphosphatemia, anxiety, and emotion-oriented coping with stress independently led to the pathogenesis of restless leg syndrome (RLS) in patients undergoing haemodialysis. The altered dopaminergic system under RLS led to disturbed excretion of phosphate [65]. In hyperphosphatemia, diffused vascular calcification occurs in patients with end-stage renal failure; this may lead to the deposition of calcified products in vasculatures of the leg and result in RLS development.

### 5.3. Skin Disorders

After a renal transplant, the prescribed immunosuppressants are absorbed into the skin and subsequently weaken the immune system, thereby creating a favourable environment for opportunistic skin infections. The most common symptom of fungal skin infection reported among patients with renal disease is pale/dark patches or liverish-looking spots. Another common skin infection among patients with renal disease is warts, which are caused by viral infections and can easily spread throughout the body. In addition, if the wart is caused by papillomavirus, it could be carcinogenic and may develop into skin cancer under UV-C-containing sunlight [66].

Among the unusual but severe calcification-related kidney complications, calciphylaxis is generally reported in patients with renal disease and advanced CKD (hyperphosphatemia), dialysis, or kidney transplants. Skin lesions in calciphylaxis are found in high-fat areas like breasts, buttocks, and abdomen [67].

The understudied diseases related to CKD should be included by evaluating comprehensive strategies to target multiple issues at a time. However, the current treatment options are scarce and limited by side effects. Therefore, we must develop safer and potentially effective alternatives to manage hyperphosphatemia appropriately.

## 6. Biological Approach for CKD and the Associated Hyperphosphatemia

To the best of our knowledge, prime investigations or attempts to explore a biological approach for CKD prevention are yet to be initiated. The primary demand for safer biological approaches for the prevention of hyperphosphatemia necessitates the screening of probiotics or intestinal bacteria as potential phosphate-accumulating organisms (PAO). Few attempts have been made with probiotic cultures to reduce uremic toxins and cure CKD; for example, oral administration of *Lactobacillus acidophilus* for 1–6 months resulted in decreased serum dimethylamine and nitrosodimethylamine (potent uremic toxins) [42]. *Oxalobacter formigenes* was administered to remove accumulated oxalates in patients with urolithiasis [68].

### 6.1. Screening and Isolation of PAOs

We previously identified *Lactobacillus casei* JCM 1134 and *Bifidobacterium adolescentis* JCM 1275 as potential PAOs [69]. However, these strains were found to be contaminated with non-PAOs, thereby decreasing the total efficiency of phosphate accumulation. To this end, we developed a screening and isolation method to obtain superior PAOs from *L. casei* JCM 1134 and *B. adolescentis* JCM 1275 by enrichment in phosphate-rich media and then eliminating non-PAOs in a low-pH selection medium by utilising the low pH survival strategy of PAOs. This was followed by the purification of cultures via centrifugation on a Percoll density gradient. Later, a novel semiquantitative assay on toluidine blue agar and a quantitative microbial-phosphate estimation method [70] were developed to detect the potential of PAOs as phosphate accumulators. These methods specifically remove the ambiguities due to interfering agents and use specific blanks for accurate phosphate estimations. The experimental data demonstrated that using strain 11th isolate of *L. casei* JCM 1134 and strain 8th isolate of *B. adolescentis* JCM 1275 as potential phosphate accumulators can be a safe and promising approach to prevent CKD-associated hyperphosphatemia in the early stages of development [69]. Furthermore, these potential PAOs were delivered as functional foods (synbiotics) to obtain high throughput product development to prevent hyperphosphatemia.

### 6.2. Functional Food Formulation and In Vitro Studies

A tailored double-layered synbiotic formulation was engineered by spray-drying the transformed phosphate-deficient *L. casei* JCM 1134 with the supernatant of lysine derivative of *Aloe vera* as an outer sacrificial layer to stand with gastric juice; then, the inner second layer of probiotic, which is a lysine derivative of *A. vera*, would selectively capture phosphate ions for the accumulation in the core (probiotic) [70]. We found that the synbiotic formulation promoted better phosphate removal when compared to that promoted by lanthanum carbonate and aluminium hydroxide. Moreover, the synbiotic formulation demonstrated efficient phosphate removal relative to the calcium carbonate from milk and soft drinks. Overall, the synbiotic formulation stands out as the first preference for phosphate removal from phosphate-rich synthetic media (15.7 mg dipotassium monohydrogen phosphate salt/mL) under simulated in vitro conditions [71].

## 7. Conclusions

Besides being expensive, current treatment methods like chemotherapy and dialysis have long-term side effects. Medical doctors prescribe phosphate binders to treat hyperphosphatemia and target only the blood phosphate levels. However, researchers and medical doctors should understand the phosphate metabolism associated with health-hazardous pathways (especially the understudied diseases) and provide comprehensive treatment by targeting probable hyperphosphatemia.

The available phosphate binders have a high pill burden and cost and lead to heavy metal deposition, metabolic irregularities, and vascular calcifications. New and safer phosphate binders are still under investigation and will not be available soon to patients currently affected by CKD. Although renal treatment strategies are prescribed in the subsequent stages, the reversal of kidney function is almost impossible, and this ensures that preventive strategies are better than treatment. In this regard, using probiotics as PAOs and their utilisation as phosphate accumulators under gastric systems is a new and safer approach to prevent hyperphosphatemia.

## 8. Future Directions

Probiotics are a safe potential option and confer additional health benefits to the gut. Prevention of hyperphosphatemia using probiotics as PAOs still needs further investigation, including extensive in vivo studies to obtain a final product that can be considered satisfactory by patients with CKD. These novel synbiotics can be incorporated into phosphate-rich foods generally barred for patients with renal disease, thereby enhancing the functional food market.

It has been reported that a phosphate-restricted life and a tight dialysis schedule have worsened the quality of life of patients. However, patients with renal disorders are still unaware of the phosphate content in natural and commercial food items and require the guidance of a good dietician. Furthermore, governments must take action to make phosphate content marking on food items obligatory in the list of ingredients. Researchers must explore the incidence of cross-reactivity other than Maillard reactions during food processing and evaluate the impact of this on renal health. In addition, government agencies must conduct social programs to spread awareness regarding the hazardous effects of phosphate and their prevention.

## Figures and Tables

**Table 1:** Advantages and disadvantages of commercially available phosphate binders [46,47,48,49,50,51,52,53,54,55].

Phosphate Binders	Dosage (per Day)	Advantages	Disadvantages
Calcium carbonate	500–600 mg, 3 times	Relatively inexpensive and first-line treatment	Can potentially lead to hypercalcemia, cardiovascular disorder (CVD), gastrointestinal risks, and vascular and bone calcification
Calcium citrate	4.5 g	Cost-effective	Can potentially lead to hypercalcemia and enhanced intestinal aluminium absorption
Calcium acetate	667–6000 mg, 9 times	Less calcium than calcium carbonate	Needs prescription
Aluminium hydroxide	600–1200 mg, 3 times	No calcium and effective across a wide range of pH levels	Can potentially lead to aluminium deposition into bones and requires strict monitoring of aluminium levels, and haematological and neurological toxicity
Lanthanum carbonate	0.5–1 g, 3 times	No calcium and effective across a wide range of pH levels	Expensive, there is deposition into bones, and can lead to toxicity, muscular-ache, and gastrointestinal risks
Magnesium carbonate	-	Effective and relatively inexpensive	Gastrointestinal risks and requires strict monitoring of magnesium levels
Sevelamer oxyhydroxide	0.5–3 g, 3 times	Effective across a wide range of pH levels, no calcium and lanthanum, reduces low-density cholesterol, and minimal assimilation	Expensive, has gastrointestinal risks, can lead to metabolic acidosis and hinder the assimilation of fat-soluble vitamins
Sucroferric oxyhydroxide	500 mg, 3 times	Effective with low pill burden	Gastrointestinal risks and expensive
Ferric citrate	210 mg, 9 times	Effective for iron and phosphate parameters	Expensive
Nicotinamide	1.5 g	Effective with low pill burden and treats pellagra	Exacerbates hyperuricemia and can lead to nausea and hyperglycaemia
Tenapanor	30 mg, 2 times	Effective with low pill burden and treats constipation	Diarrhoea and nausea

## Data Availability

All the data analysed is included in this article.

## Supplementary Materials

The following supporting information can be downloaded at: https://www.mdpi.com/article/10.3390/medicina59050959/s1, Renal damage caused by heavy metals in cosmetics and SARS-CoV-2 (separate text file). Refs. [72,73,74,75,76,77,78,79,80,81,82,83,84,85,86].
