# Supplementary material for: Understudied Hyperphosphatemia (Chronic Kidney Disease) Treatment Targets and New Biological Approaches
Source: Medicina (Kaunas). 2023 May 16;59(5):959. doi: 10.3390/medicina59050959 (PMC10221414; doi:10.3390/medicina59050959)
Supplement: Supplementary file 1 [file medicina-59-00959-s001.zip › medicina-2263900-supplementary.pdf]

Special note for attention required renal related topics

Renal damage causing hard metals in cosmetics

I would like to brief about the renal damaging hard metals used in cosmetics as cosmetics are in our daily routine and might probably lead to hyperphosphatemia, even though it is little out of scope for this article. Cosmetics are at high consumption demand around the world, especially for women. Cadmium and lead are commonly used in the cosmetics industry for their attractive color pigments, although they are present around few micrograms per gram of soap, shaving cream, face cream, shampoo, and lipstick. But publications established the long-term usage of cadmium and lead may cause accumulation in kidneys and led to nephropathy or liver damage (determination of lead and cadmium in cosmetics). The other effects of cadmium poisoning are nausea, vomiting, diarrhoea, headache, respiratory distress, and bronchopneumonia. While lead poisoning may lead to ataxia, headache, vomiting, stupor, hallucination, tremors, and convulsions. The chronic exposure of lead and cadmium in conjunction may result in loss of cognitive function, renal damages, and memory loss. Surprisingly, no medical practitioner or cosmetics industries talks about the harmful effects of lead and cadmium present in cosmetics for renal damage [s1].

Renal damage caused by SARS CoV-2

According to literature, kidneys are the common target of corona infection. The comorbidities with diabetes, hypertension and immunosuppressant therapies cause the disbalance in immune system and resulted to kidney damage [s2,s3]. Corona patients with kidney disorder and obesity are at higher risk for morbidity and mortality due to the probable multi-organ damages. Medical practitioners must be cautious to prescribe the appropriate medical therapies to not worsen the kidney damage of CKD patients with corona infection as well as close monitoring and the screening of other opportunistic infections must be routinely tested to ensure the proper management and reduce the further renal injuries. Recent studies corroborated the statistics of 2 % of corona patients have CKD [s4]; in addition, the renal transplant patients are at higher risk for corona infection due to the prescription of immunosuppressants [s5]. Moreover, acute kidney injury is the common factor for the severity and mortality among corona patients [s6]. As per recent statistics, severe corona infection is found around 83 % among 1 % of CKD patients, that caused the 53 % mortality for the corona-renal patients [s7]. In other reports, the mortality rate of CKD patients with corona was found to be 60-90 % [s8,s4,s9]. According to Cheng et al, about 3.2 % of developing corona patients progressed to kidney damage [s4].

In a retrospective study, it was found that corona virus damages the renal tubules and acute renal failure occurs with aged and co-morbid corona patients [s10]. Corona virus accelerates the C-reactive protein and enhances the neutrophil ratio by several folds that causes the inflammation and cytotoxicity in renal tubules [s11]. Kidney damage in corona patients can be early screened by the reduction in GFR, creatinine clearance and the development of microalbuminuria [s11]. The kidney damage in corona patients is evident by a postmortem study from China that revealed the "diffused acute proximal tubular injury with loss of brush border and non-isometric vacuolation" [s12]. These mentioned renal damages and podocytes inflammation are also published as linkage with proteinuria and acute kidney injury that was supported by Larsen et al [s3]. Acute tubular necrosis, interstitial inflammation, renal vasculature, and glomerulus damages are the prominent causes of acute and chronic kidney damages. At early stages of COVID-19, endothelial dysfunctions are reported such as pericyte detachment,

elongation in sub-endothelial space and foam cell development [s13]. Additionally, due to the endothelial damages, vasoconstriction, ischemia, and inflammation occurs that lead to hypoxia related renal damage and pulmonary oedema and bleeding disorders [s14].

In a study, it was suggested that the haemodialysis and renal transplant patients may not develop the severe corona conditions due to the impaired immune system as immunosuppressants are especially prescribed to them that may hinder the hyper acceleration of immune response as cytokine storm [s15]. However, in such patients, the close monitoring of oxygen levels and lungs functionality must be in care as their immunity is in suppressive mode and patients can be asymptomatic to diagnose the corona infection. For the treatment of renal transplanted patients with corona, mild dose of methylprednisolone-based therapy is recommended [s16].

Supplementary references

S1. Skin care in renal transplant patients _ National Kidney Federation. https://www.kidney.org.uk/skin-care-in-renal-transplant-patients. Accessed July 8, 2021.

S2. Perico L, Benigni A RG. Should COVID-19 concern nephrologists? Why and to what extent? The emerging impasse of angiotensin blockade. *Nephron*. 2020;144:213-221.

S3. Larsen CP, Bourne TD, Wilson JD, Saqqa O SM. Collapsing glomerulopathy in a patient with coronavirus disease 2019 (COVID-19). *Kidney Int Rep*. 2020;5:935-939.

S4. Cheng Y, Luo R, Wang K, Zhang M, Wang Z, Dong L et al. Kidney impairment is associated with in-hospital death of COVID-19 patients. *medRxiv*. 2020;97:829-838.

S5. Banerjee D, Popoola J, Shah S, Ster IC, Quan V PM. COVID-19 infection in kidney transplant recipients. *Kidney Int*. 2020;97:1076-1082.

S6. Huang C, Wang Y, Li X, Ren L, Zhao J, Hu Y et al. Clinical features of patients infected with 2019 novel coronavirus in Wuhan, China. *Lancet*. 2020;395:497-506.

S7. Oyadela T, Alqahtani J CG. Prognosis of COVID-19 in patients with Liver and Kidney diseases: an early systemative review and meta-analysis. (2020) 5:1–14. *Trop Med Infect Dis*. 2020;5:1-14.

S8. Chen N, Dong X, Qu J, Gong F, Han Y, Qiu Y et al. Epidemiological and clinical characteristics of 99 cases of 2019 novel coronavirus pneumonia in Wuhan, China: a descriptive study. *Elsevier Enhanc Reader Lancet Lond Engl*. 2020;395:507-513.

S9. Dawei W, Bo H, Chang H, Fangfang Z, Xing L, Jing Z et al. Clinical characteristics of 138 hospitalized patients with 2019 novel coronavirus- infected pneumonia in Wuhan, China. *JAMA*. 2020;7323:1061-1069.

S10. Diao B, Wang C, Wang R, Feng Z, Tan Y, Wang H et al. Human kidney is a target for novel severe acute respiratory syndrome coronavirus 2 (SARS-CoV- 2) infection. *medRxiv [Preprint]*. 2020.

S11. Hong X, Chi Z, Liu G, Huang H, Guo S, Fan J et al. Analysis of early renal injury in COVID-19 and diagnostic value of multi-index combined detection. *medRxiv [Preprint]*. 2020.

S12. Su H, Yang M, Wan C, Yi, L.-X., Tang F, Zhu, H.-Y. et al. Renal histopathological analysis of 26 postmortem findings of patients with COVID-19 in China. *Elsevier Enhanc Reader Kidney Int*. 2020;98:219-227.

S13. Pan X, Xu D, Zhang H, Zhou W, Wang L CX. Identification of a potential mechanism of acute kidney injury during the COVID-19 outbreak: a study based on single-cell transcriptome analysis. *Intensive Care Med*. 2020;46:1114-1116.

S14. Khoshdelrad N, Zahmatkesh E, Shpichka A, Timashev P VM. Outbreak of chronic renal failure: will

this be a delayed heritage of COVID-19? *J Nephrol*. 2020;2:1-3.

S15. Ma Y, Diao B, Lv X, Zhu J, Liang W, Liu L et al. COVID-19 in hemodialysis (HD) patients: report from one HD center in Wuhan, China. *medRxiv*. 2020;98:17-19.

S16. Zhu L, Xu X, Ma K, Yang J, Guan H, Chen S et al. Successful recovery of COVID-19 pneumonia in a renal transplant recipient with long-term immunosuppression. *Am J Transplant*. 2020;20:1859-1863.
